# Physical activity and sedentary behavior surveillance using accelerometers in Japanese urban adults: A descriptive study of participation and adherence

**DOI:** 10.1371/journal.pone.0350144

**Published:** 2026-06-01

**Authors:** Naruki Kitano, Ryoko Kawakami, Yuya Fujii, Daisuke Yamaguchi, Yuko Muramatsu, Yuki Matsushita, Yosuke Mizuno, Sachiko Miyamoto, Tomohiko Yoshida, Yuko Kai, Takashi Arao

**Affiliations:** 1 Physical Fitness Research Institute, Meiji Yasuda Life Foundation of Health and Welfare, Tokyo, Japan; 2 Sasakawa Sports Foundation, Tokyo, Japan; University of Auckland, NEW ZEALAND

## Abstract

**Background:**

Representative data collection in accelerometer-based physical activity and sedentary behavior surveys is challenging. Although in-person protocols and monetary incentives are recommended to enhance recruitment, the participation outcomes of such methods have not been thoroughly examined. This study aimed to (1) quantify response and adherence rates for an in-person, incentivized accelerometer survey; (2) assess participant representativeness; and (3) characterize reasons and factors associated with participation.

**Methods:**

This cross-sectional study randomly selected 650 Japanese adults aged 20–79 from resident registers in three major metropolitan areas. Trained investigators delivered and retrieved accelerometers and questionnaires. A 5000-yen incentive was provided upon completion. Primary outcomes were response and adherence rates (≥ 4 valid wear days). Participants’ representativeness was evaluated by comparing sociodemographic characteristics with national census data, and reasons for participation and non-participation were summarized.

**Results:**

The response rate was 31.5%, and the adherence rate was 29.1%. Compared to census data, participants were older and more likely to be employed and living with others; however, gender distribution was comparable. Motivations for participation differed by age: older adults were mainly motivated by health-consciousness, whereas younger adults cited the monetary incentive. Non-contact at home was a major reason for non-participation among younger adults.

**Conclusions:**

The in-person, incentivized protocol achieved a higher response rate than typical mail-based methods in Japan and produced a gender-balanced sample, although some selection bias remains. These findings suggest that while this resource-intensive protocol can enhance overall recruitment, future strategies tailored to participant sociodemographic characteristics are warranted to maximize accelerometer-based data collection.

## Introduction

There is a growing need to develop public health strategies to address the global burden of disease caused by insufficient physical activity (PA) and prolonged sedentary behavior (SB) [1]. Population-based surveys are essential for understanding the prevalence and determinants of physical inactivity and for evaluating the impact of related interventions and policies. In this context, device-based measurements such as accelerometers are preferred over self-reported assessments, as they not only provide more detailed and comprehensive data on PA but also offer greater reliability and validity [2–4]. Owing to these strengths, accelerometers are now widely employed in epidemiological studies.

However, in epidemiological research, achieving a representative sample in device-based studies is challenging because the burden of wearing the device for an extended period may discourage participation. Low participation and adherence can introduce bias, compromising both internal and external validity [5–7]. Previous studies have shown that participants in accelerometer-based surveys are often not fully representative of the source population, tending to differ in characteristics such as age, sex, health status, and health-related behaviors [8–11]. Prior research has also suggested that participation and adherence may be influenced by factors such as perceived burden, health interest, and study procedures [9,11]. Therefore, identifying strategies to enhance recruitment and participation in population-based accelerometer surveys remains a significant public health concern.

Recently, the first systematic review of observational studies using accelerometers was published [11]. This review included 95 studies and summarized overall response rates, adherence to valid wear protocols, and methodological factors associated with successful data collection [11]. The average response rate was reported as 75.0%, and in-person distribution of devices was associated with higher participation and adherence (i.e., meeting valid wear-time criteria). The review also recommended the use of monetary incentives as an effective strategy to enhance response rates.

Nevertheless, several knowledge gaps remain. First, many of the reviewed studies recruited participants who had already been involved in prior surveys or health examinations, rather than sampling directly from the general population. Such individuals may be more inclined to participate in additional accelerometer-based data collection, thereby overestimating response rates. Second, methodological factors influencing participation and adherence may vary across countries, populations, and survey settings [11]. Therefore, findings from international studies may not be directly generalizable to the Japanese context, underscoring the need for further research to assess their applicability domestically. To date, only five studies have evaluated participation and adherence in population-based accelerometer surveys among Japanese adults (see S1 Table) [10,12–15]. However, all of these studies used a mail-based approach for device distribution and retrieval. Thus, although in-person protocols are generally considered preferable for maximizing data collection, their influence on response rates and associated factors has not been sufficiently examined in Japan.

To address these gaps, we conducted a population-based survey of Japanese adults to assess PA and SB using accelerometers, with in-person distribution and retrieval of devices and monetary incentives. Based on previous findings that accelerometer study participants tend to be older, female, and healthier than the source population [8–11], we were particularly interested in whether similar patterns of selection bias emerged under our in-person, incentivized protocol. The aims of this descriptive study were threefold: (1) to quantify response and adherence rates; (2) to examine the participant representativeness by comparing their sociodemographic characteristics with national census data; and (3) to characterize the reasons and factors associated with participation and non-participation. Our findings are expected to inform the design of efficient and practical protocols for future device-based surveys.

## Materials and methods

This study adhered to Strengthening the Reporting of Observational Studies in Epidemiology (STROBE) guidelines (S2 Table).

### Participants

This cross-sectional, population-based survey utilizing accelerometers was conducted in three major metropolitan areas of Japan in October 2023. Participants were Japanese adults aged 20–79 years, randomly selected through stratified sampling from the Basic Resident Registers. Specifically, participants were selected from three metropolitan regions encompassing 13 prefectures: Tokyo (Tokyo, Kanagawa, Chiba, Saitama), Chukyo (Aichi, Gifu, Mie), and Hanshin (Osaka, Kyoto, Nara, Hyogo, Wakayama, Shiga). These regions were further stratified into four categories based on population size: towns and villages, cities with fewer than 100000 residents, cities with 100000 or more residents, and the special wards of Tokyo and ordinance-designated cities. This stratification yielded 12 divisions (3 regions × 4 city sizes). Sampling quotas were proportionally allocated to each division based on population size, and participants were recruited from 50 sites (cities, towns, and villages). From each site, 13 individuals were randomly selected from the Basic Resident Registers, yielding a total of 650 invited participants. Written and oral informed consent was obtained from all participants. This study protocol was approved by the Ethics Committee of the Physical Fitness Research Institute, Meiji Yasuda Life Foundation of Health and Welfare (approval number: 2023−0004).

### Surveillance methods

Data collection followed a drop-off/pick-up method, in which trained investigators visited the homes of potential participants to deliver the accelerometer and questionnaire and later returned to retrieve them. The recruitment period was from October 21 to November 12, 2023. Prior to the home visit, each potential participant received a postcard notifying them that a trained investigator would visit their home during a specified period. If the participant was not available during the initial visit, investigators made up to 11 additional visits (mean: 3.2 visits per participant) to establish contact. Investigators (*n* ≈ 50) visited participants’ homes, explained the study procedures, and asked them to wear an accelerometer and complete a questionnaire. As an incentive for participation, respondents received (1) a 5000-yen QUO card (a prepaid gift card widely used in Japan), provided upon completion of the study protocol when investigators returned to collect the materials, and (2) personalized feedback on their PA and SB results, upon request. The conditional incentive was designed to encourage both initial participation and adherence to the accelerometer wear protocol. Several weeks later, investigators returned to collect the materials and deliver the incentive. A call center was available throughout the survey period to respond to participant inquiries.

### Nationally representative data used to assess the representativeness of study participants

To assess the representativeness of participants who completed the survey, we compared their sociodemographic characteristics with data from the 2020 Japanese Population Census, conducted by the Ministry of Internal Affairs and Communications [16]. The census, conducted every five years, includes all Japanese residents. We used publicly available summary data from the most recent census (2020). Specifically, we extracted data corresponding to the population targeted in this study—individuals aged 20–79 years residing in the same 50 sites—and focused on variables comparable to those collected in our survey: age, gender, employment status, living arrangement, and educational attainment. For comparison of mean age, we calculated a population-weighted mean age based on census data from the selected sites.

### Measurements

#### Sociodemographic characteristics.

Age and gender data for all 650 invited participants were obtained from the Basic Resident Registers. To describe respondent characteristics, additional data were collected via a self-administered questionnaire, including living arrangement, employment status, job type, educational attainment, annual household income, height, weight, and exercise habits. Body mass index (BMI) was calculated as weight (kg) divided by height (m) squared.

#### Physical activity and sedentary behavior.

PA and SB were assessed using a research-grade tri-axial accelerometer (Active Style Pro HJA750-C; Omron Healthcare, Kyoto, Japan). This device has been previously validated [17], and its measurement accuracy is comparable to that of accelerometers commonly used in Western countries [18,19]. Participants were instructed to wear the device on their waist during waking hours for at least seven consecutive days, including weekends, except during activities that could damage the device (e.g., water-based activities or contact sports). The device screen did not display PA information during the assessment period. The epoch length was set at 60 seconds, and metabolic equivalents (METs) were estimated from synthetic acceleration data using manufacturer-provided software [20]. Non-wear time was defined as at least 60 consecutive minutes of activity counts below the detection threshold. A valid day was defined as having at least 10 hours of wear time [21]. Based on estimated METs, each 60-second epoch was classified as SB (≤ 1.5 METs), light-intensity PA (LPA; 1.6–2.9 METs), or moderate-to-vigorous PA (MVPA; ≥ 3.0 METs), following previous studies [22,23]. Daily values were aggregated and averaged across all valid days. Participants with at least four valid days, including at least one weekend day, were defined as adherent and included in the analysis [24]. We also assessed the proportion of participants meeting Japan’s national PA guidelines: ≥ 60 minutes per day of MVPA for adults aged 20–64 years, and ≥ 40 minutes per day for those aged ≥ 65 years [25].

#### Reason for (non)participation in the survey.

Investigators recorded the following reasons for nonparticipation during their visits: (1) absence (i.e., no one was at home or appeared to be intentionally unavailable); (2) refusal (e.g., citing inconvenience or being too busy); and (3) inability to participate due to issues with the accelerometer (e.g., injury, illness, long-term hospitalization or travel) or the questionnaire (e.g., cognitive decline or language barriers). In the questionnaire, participants indicated their reasons for participation by selecting from the following options (multiple responses allowed): (1) interest in their own PA and SB; (2) the investigator’s clear and thorough explanation; (3) desire to contribute to society; (4) general health consciousness (i.e., attention to one’s own health and lifestyle); (5) the participation incentive; and (6) other.

### Statistical analysis

For the first objective, we calculated the response rate (i.e., the proportion of invitees who agreed to participate) and the adherence rate (i.e., the proportion who met valid wear criteria) for this survey.

For the second objective, which involved evaluating the representativeness of participants, we summarized participants’ sociodemographic characteristics and lifestyle factors using means and standard deviations (SDs) for continuous variables and proportions for categorical variables. PA and SB variables were summarized using medians and interquartile ranges due to their non-normal distributions. Raincloud plots (combining boxplots, half-violin plots, and scatter plots) were used to visualize the distributions of PA and SB. Comparisons with the source population were limited to age, sex, and residential area, which were available from the Basic Resident Registers. When available, participant characteristics were also compared with those in the national census.

For the third objective, we summarized the distribution of reported reasons for participation and non-participation. Due to the small sample size, participants who selected “other” as a reason for participation (e.g., citing low burden) were excluded from the analysis. To adjust for potential confounding and enhance interpretability, we conducted multivariable analyses to examine associations of sociodemographic characteristics with participation, adherence, and reasons for participation or nonparticipation. Modified Poisson regression models were used to estimate prevalence ratios (PRs) and their 95% confidence intervals (CIs) [26]. In these models, participation, adherence, and reasons for participation or nonparticipation were treated as dependent variables, while age (20–39, 40–59, 60–79 years), gender (men or women), and residential population size (< 100000; 100000–299999; ≥ 300000) were simultaneously included as independent variables. Statistical significance was set at *P* < 0.05. All analyses were conducted using R version 4.4.2 (R Foundation for Statistical Computing, Vienna, Austria).

## Results

### Response and adherence rate to the survey

**Fig 1:**
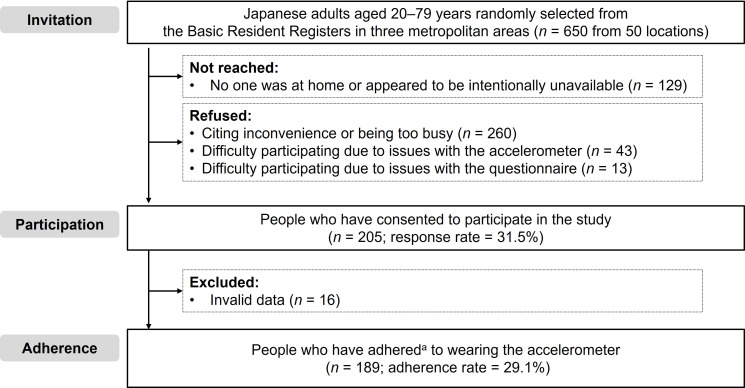
Selection flow of the study participants. ^a^At least four valid days, including at least one weekend.

The participant selection flow is presented in Fig 1. Of the 650 individuals invited, 129 (19.8%) could not be reached because no one was at home or they appeared to be intentionally unavailable. Among the remaining individuals, 260 (40.0%) declined participation, reporting inconvenience or being too busy; 43 (6.6%) cited difficulty wearing the accelerometer, and 13 (2.0%) cited difficulty completing the questionnaire. Ultimately, 205 individuals completed both the accelerometer protocol and the questionnaire, yielding a response rate of 31.5%. Finally, 189 participants met the valid wear-time criteria (≥ 4 days, including at least one weekend day), with an adherence rate of 29.1%.

### Characteristics of the study participants

Table 1 presents the characteristics of invited, participating, and adherent individuals, with comparisons to the 2020 Japanese Population Census. Among adherent participants, the mean (SD) age was 53.0 (14.0) years; 49.2% were women, 83.5% were working (including part-time and self-employed individuals), and 57.6% had a college degree or higher. Compared to census respondents, participants in this study were older (53.0 vs. 49.5 years), more frequently employed (83.5% vs. 57.4%), and less likely to live alone (14.3% vs. 21.8%). No substantial differences were observed in gender or educational attainment. Multivariable analysis supported these patterns: older individuals (aged 60–79 years) were more likely to participate (PR = 1.79, 95% CI = 1.30–2.46 for 40–59 years; PR = 1.44, 95% CI = 1.01–2.05 for 60–79 years) and to meet the valid wear-time criteria (PR = 2.00, 95% CI = 1.41–2.86 for 40–59 years; PR = 1.65, 95% CI = 1.12–2.42 for 60–79 years), compared to younger individuals (S3 Table).

**Table 1:** Characteristics of study participants compared with respondents in the 2020 Japanese population census.

Variables	Invited (*n* = 650)	Consented (*n* = 205)	Adhered (*n* = 189)	Population Census 2020
Region				
Tokyo metropolitan area	338 (52.0%)	93 (45.4%)	86 (45.5%)	NA
Hanshin metropolitan area	195 (30.0%)	58 (28.3%)	54 (28.6%)	NA
Chukyo metropolitan area	117 (18.0%)	54 (26.3%)	49 (25.9%)	NA
Population				
≥ 300,000	208 (32.0%)	62 (30.2%)	56 (29.6%)	NA
100,000–299,999	286 (44.0%)	81 (39.5%)	75 (39.7%)	NA
< 100,000	156 (24.0%)	62 (30.2%)	58 (30.7%)	NA
Mean (SD) age, years	51.0 (16.0)	52.3 (14.3)	53.0 (14.0)	49.5
Women	308 (47.4%)	99 (48.3%)	93 (49.2%)	4328539 (50.1%)
Employment status				
Full-time worker	NA	121 (59.3%)	112 (59.6%)	4308033 (49.1%)
Part-time worker	NA	48 (23.5%)	45 (23.9%)	741279 (8.3%)
Homemaker	NA	19 (9.3%)	18 (9.6%)	1028493 (11.5%)
Student	NA	0 (0.0%)	0 (0.0%)	140764 (1.6%)
Unemployed	NA	14 (6.9%)	12 (6.4%)	1036919 (11.6%)
Others/Unknown	NA	2 (1.0%)	1 (0.5%)	1692722 (18.9%)
Living alone	NA	31 (15.1%)	27 (14.3%)	1932458 (21.8%)
Education				
≤ High school	NA	87 (43.5%)	78 (42.4%)	2974060 (43.6%)
College	NA	38 (19.0%)	37 (20.1%)	1268996 (18.6%)
University or graduate school	NA	75 (37.5%)	69 (37.5%)	2575958 (37.8%)
Household income				
< 3 million yen	NA	44 (25.1%)	41 (25.3%)	NA
≥ 3 million yen	NA	32 (18.3%)	31 (19.1%)	NA
≥ 5 million yen	NA	24 (13.7%)	23 (14.2%)	NA
≥ 7 million yen	NA	27 (15.4%)	24 (14.8%)	NA
Unknown or do not want to answer	NA	48 (27.4%)	43 (26.5%)	NA
Mean (SD) BMI, kg/m^2^	NA	22.7 (3.4)	22.6 (3.2)	NA
Exercise habit	NA	38 (18.6%)	37 (19.7%)	NA

BMI, body mass index; NA, not available; SD, standard deviation.

**Fig 2:**
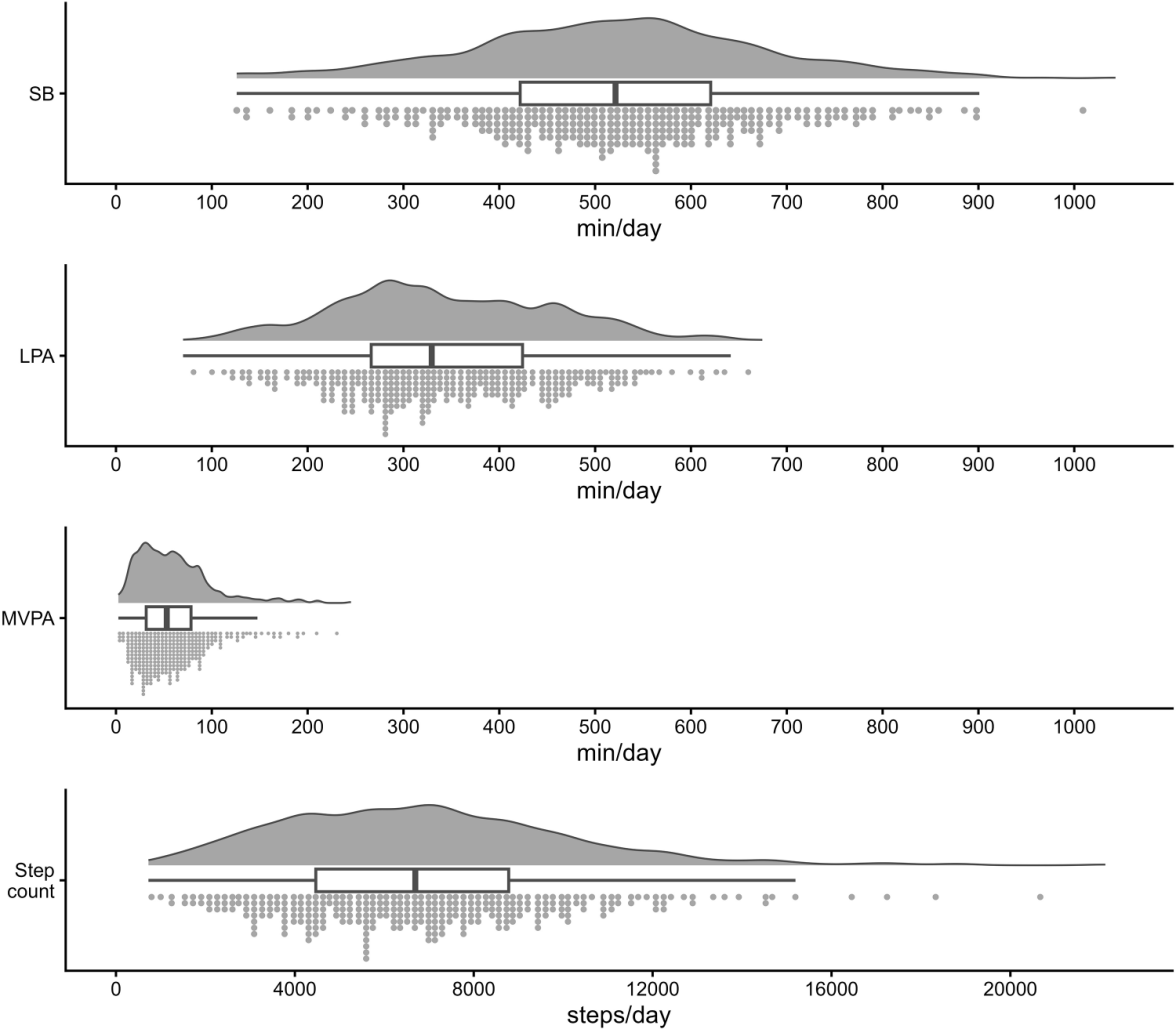
Description of accelerometer-measured physical activity and sedentary behavior among adherent participants (*n* = 189). LPA, light-intensity physical activity; MVPA, moderate-to-vigorous physical activity; SB, sedentary behavior.

Descriptive statistics for accelerometer-measured PA and SB are presented in Fig 2 and S4 Table. The median (range) values were 7 days (4–14) for valid wear days, 529.9 minutes/day (135.5–900.9) for SB, and 6925 steps/day (1299–18853) for step count. The proportion of participants meeting the PA guidelines set by Japan’s Ministry of Health, Labour and Welfare was 50.3%. When expressed as a proportion of wear time, younger adults (20–64 years) spent more time in MVPA than older adults (≥ 65 years) (6.2% vs. 5.5%), whereas time spent in SB (57.4% vs. 57.9%) and LPA (36.0% vs. 37.1%) was comparable between age groups. Men spent more time in SB (60.9% vs. 52.3%) and less in LPA (32.2% vs. 41.0%) compared to women. Given the absence of comparable national reference data, comparisons of PA distributions with nationally representative surveys were not possible.

### Reasons for the non-participation and participation

**Fig 3:**
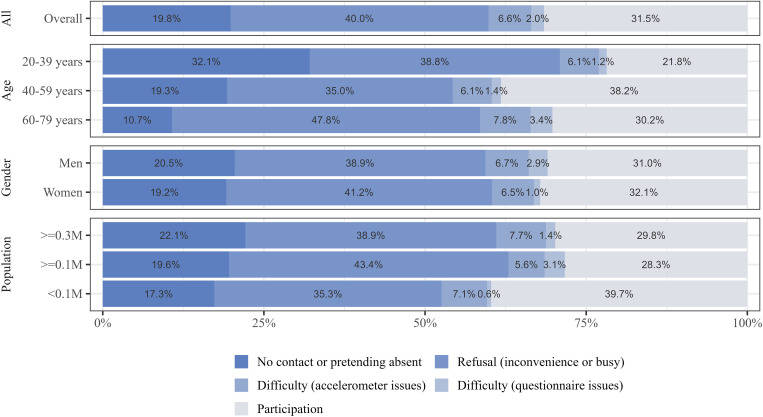
Reasons for non-participation by sociodemographic characteristics among all invited participants (*n* = 650). The unit of population is a million.

Fig 3 illustrates the reasons for non-participation among all invitees, stratified by sociodemographic characteristics. The proportion of non-participation classified as non-contact (i.e., being absent or unreachable) was notably lower in older age groups compared to the youngest group (10.7–19.3% for ages 40–79 vs. 32.1% for ages 20–39). This age-related pattern was consistent with multivariable analysis, which showed significantly lower prevalence ratios for non-contact in older age groups (PR = 0.59, 95% CI = 0.43–0.82 for ages 40–59; PR = 0.33, 95% CI = 0.21–0.52 for ages 60–79; S5 Table). No notable differences in reasons for non-participation were observed by gender or residential population size.

**Fig 4:**
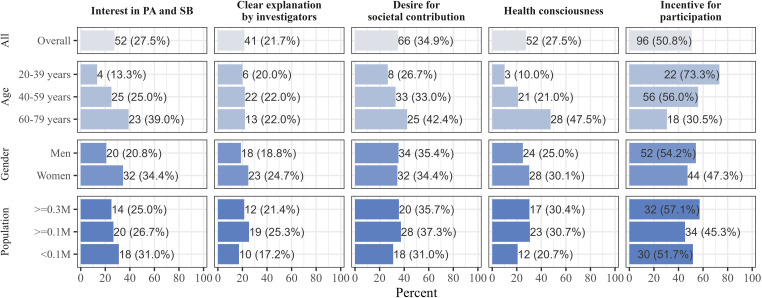
Reasons for survey participation by sociodemographic characteristics among adherent participants (*n* = 189). The unit of population is a million. PA, physical activity; SB, sedentary behavior.

Fig 4 presents the reasons for participation among adherent participants. The most frequently reported reasons were the monetary incentive (50.8%), contribution to society (34.9%), interest in personal PA and SB (27.5%), general health consciousness (27.5%), and the investigator’s clear and thorough explanation (21.7%). Multivariable analysis showed that older adults (ages 60–79) were more likely than younger adults to report interest in their own PA and SB (PR = 3.27, 95% CI = 1.22–8.81) or general health consciousness (PR = 4.72, 95% CI = 1.55–14.39) as their participation motivation. Conversely, they were less likely to cite the monetary incentive (PR = 0.41, 95% CI = 0.26–0.65). Furthermore, women were more likely than men to cite interest in their own PA and SB as a reason for participation (PR = 1.77, 95% CI = 1.11–2.82; S6 Table). Conversely, women were less likely than men to cite the monetary incentives, although this difference was not statistically significant (PR = 0.81, 95%CI = 0.61–1.06).

## Discussion

We conducted a population-based survey among general Japanese adults using accelerometers, in-person device distribution and retrieval, and monetary incentives. The resulting response and adherence rates were 31.5% and 29.1%, respectively. Compared with national census data, study participants were more likely to be older, employed, and living with others, although no substantial differences were observed in gender or educational attainment. Given the absence of nationally representative data on device-measured PA and SB, we could not compare our findings with national benchmarks. We also observed that reasons for participation and non-participation varied by sociodemographic characteristics, providing insights for refining future data collection strategies. While the descriptive nature of this study warrants caution in interpreting these findings, the results indicate several points.

First, our protocol-combining in-person device distribution and retrieval with a relatively high monetary incentive may have contributed to the higher response rate observed, relative to prior mail-based surveys. According to previous studies in Japan, mail-based accelerometer surveys of adults randomly selected from the Basic Resident Registers have yielded response rates of 13% to 23% [10,12–15], while those offering monetary incentives have reported rates of 14% to 22% [10,15]. In comparison, the response rate in our study was 31.5%. This improvement may be partially attributable to key features of our protocol, which have been suggested as determinants of participation: in-person distribution and retrieval of devices and relatively higher monetary incentives (5000 yen vs. 1000 yen) [11]. However, in adolescent populations, the influence of monetary incentives on participation in accelerometer-based surveys has been reported to be limited [27]. Further studies are needed to assess whether increasing incentive amounts can further enhance participation and to determine an appropriate cost-effective threshold.

Second, our survey protocol may help mitigate gender-related selection bias, which has been frequently reported in previous research. Previous studies have shown that, compared to the general population, participants in accelerometer-based surveys tend to be older, more likely to be women, less likely to be obese or current smokers, and report fewer health conditions [8,10,28]. In particular, age- and gender-related selection biases have been persistent challenges in this research area. However, no substantial difference in gender distribution was observed between participants in this study and the general adult population. This balanced outcome is likely attributable to two factors. First, we found no significant gender differences in reasons for non-participation, suggesting that the primary barriers to our survey were gender-neutral. Second, building on this, the study appealed to men and women through different motivational pathways: while the offer of health feedback was a significant motivator for women, this potential skew was counterbalanced by the monetary incentive, which appeared to be a more compelling reason for men. This suggests that a multi-faceted strategy combining intrinsic (e.g., feedback) and extrinsic (e.g., incentives) motivators may help mitigating gender bias. Future studies may further improve participation by tailoring recruitment messages to the motivations of specific subgroups, rather than relying solely on uniform recruitment materials.

Third, despite the resource-intensive nature of our survey method, selection bias may persist for certain sociodemographic characteristics. Specifically, participants were more likely to be older, employed, and living with others, and thus may not fully reflect the sociodemographic composition of the general population. These patterns are consistent with findings from previous studies, which report that survey participants often differ from the general population in terms of age, obesity, smoking status, and overall health [8,10,28]. We also found that younger individuals were more likely than older individuals to participate in reporting the monetary incentive. However, despite offering relatively high incentives and in-person device delivery, participation remained skewed toward older adults. These findings suggest that, even with incentives of at least 5000 yen and a drop-off/pick-up protocol, age-related participation bias may continue to affect population-based accelerometer surveys. Additionally, younger individuals were more likely to be unreachable, reporting absence or avoidance during home visits. To improve participation among younger and other hard-to-reach groups, future surveys may benefit from combining multi-modal contact strategies with appointment-based scheduling, more flexible visit times (e.g., evenings or weekends), and lower-burden options for device return or wear protocols; oversampling groups expected to have lower participation, with appropriate weighting in analysis, may also help reduce residual selection bias.

Fourth, the median daily sedentary time and step count observed in this study were approximately 530 minutes and 6900 steps, respectively. These descriptive statistics provide context for interpreting accelerometer-derived PA and SB in this population-based urban sample. Although directly comparable national benchmark data for accelerometer-derived PA and SB indicators are not available, the median daily step count in this study (7046 steps for men and 6699 steps for women) tended to be higher than the estimates from the National Health and Nutrition Survey (NHNS) conducted in the same year [29], which reported approximately 6600 steps for men and 5600 steps for women [30]. However, comparisons with the NHNS should be interpreted with caution because of methodological differences, including the short measurement period [31], lower measurement precision [32], self-reporting of recorded values [32], and possible behavioral reactivity [33]. Therefore, the generalizability of our findings should be interpreted cautiously. Nonetheless, this study provides useful descriptive data on participation rates, PA, and SB obtained using standardized survey procedures [11], including in-person device distribution and financial incentives.

This is the first study to report the participation and adherence rates of a population-based accelerometer survey incorporating in-person device distribution and retrieval alongside monetary incentives among general Japanese adults. We also assessed the representativeness by comparing the sociodemographic characteristics of the participants with those in the national census. However, this study has several limitations. First, although sociodemographic characteristics were compared with census data, lifestyle factors and health status could not be evaluated. Therefore, caution is warranted when interpreting the representativeness of the sample. Since a certain number of participants were already interested in measuring PA, we cannot rule out the influence of self-selection bias on both the participation rates and activity levels [8,34]. Second, the generalizability of our findings is limited to adults residing in Japan’s metropolitan areas. Given the sociodemographic and cultural differences between urban and rural populations, additional research is needed to evaluate whether these findings apply to the broader Japanese population, including rural residents and individuals from diverse cultural backgrounds. Third, the use of a research-grade, waist-worn accelerometer may have influenced participation and adherence. Previous studies have reported that wrist-worn accelerometers yield higher compliance with wear protocols compared to waist-worn devices [11,35]. While waist-worn devices offer advantages in classifying activity intensities [36], wrist-worn devices may lower participant burden and improve adherence. Future studies should weigh this trade-off between measurement accuracy and compliance when designing population-based surveillance protocols.

## Conclusions

This study described the participation outcomes from a population-based accelerometer survey using an in-person, incentivized protocol among Japanese adults. This approach yielded a higher response rate (31.5%) than typical mail-based surveys and achieved gender balance, although some selection bias persisted. Furthermore, the study provides valuable insights for improving future surveys by highlighting that motivations and reasons for participation vary significantly across sociodemographic groups. While resource constraints remain an important consideration, researchers and policymakers aiming to optimize accelerometer-based data collection may benefit from adopting this protocol. Further studies are needed to develop more effective data collection protocols to improve participation rates and address selection bias in population-based accelerometer surveys.

## Supporting information

S1 Table Summary of the previous studies that conducted population-based accelerometer survey in Japan. (PDF)

S2 Table STROBE Statement—Checklist of items that should be included in reports of cross-sectional studies. (PDF)

S3 Table Factors associated with participating in the survey and adherence to valid wear. ^a^Participants with at least four valid days (including one weekend). All characteristics were included in the model simultaneously. Bold values indicate *P* < 0.05.
(PDF)

S4 Table Descriptive statistics of the accelerometer-measured physical activity and sedentary behavior in the participants who adhered to the valid wear (*n* = 189). LPA, light-intensity physical activity; MHLW, Ministry of Health, Labour and Welfare; MVPA, moderate-to-vigorous physical activity; SB, sedentary behavior. ^a^ ≥ 60 min/day of MVPA for 20–64 years and ≥ 40 min/day of MVPA for 65 years and older.
(PDF)

S5 Table Factors associated with the reasons for nonparticipation in the survey among the invited sample (*n* = 650). All characteristics were included in the model simultaneously. Bold values indicate *P* < 0.05.
(PDF)

S6 Table Factors associated with the reasons for participation in the survey among the participants who adhered to the valid wear (*n* = 189). PA, physical activity; SB, sedentary behavior. All characteristics were included in the model simultaneously. Bold values indicate *P* < 0.05.
(PDF)
