# Supplementary material for: Physical activity and sedentary behavior surveillance using accelerometers in Japanese urban adults: A descriptive study of participation and adherence
Source: PLoS One. 2026 Jun 1;21(6):e0350144. doi: 10.1371/journal.pone.0350144 (PMC13225377; doi:10.1371/journal.pone.0350144)
Supplement: S1 Table — (PDF) [file pone.0350144.s001.pdf]

S1 Table. Summary of the previous studies that conducted population-based accelerometer survey in Japan.

No	Author	Survey year	Age	Gender	Survey region (prefecture)	Sampling method	Invitation method	Invitation flow	Incentives	Number of participants sampled	Number of participants	Number of adherents	Response rate	Adherence Rate	Used device	Criteria for valid wear	Details of survey protocol
1	Kondo et al.	2006	30–69 years	Men, Women	Yamaguchi	Stratified random sampling	Mail	Direct Mail	NA	480	112	112	23.3%	23.3%	Suzuken	Worn for ≥8 hours/day or with ≥50 Kcal of exercise for at least 3 days	A stratified random sample was selected from the Basic Resident Register based on age and gender, and individuals were invited to participate in the study. Those who returned the consent form and questionnaire were mailed an accelerometer.
2	Inoue et al.	2007–2008	20–69 years	Men, Women	Ibaraki, Tokyo, Shizuoka, Kagoshima	Stratified random sampling	Mail	Follow-up Invitation	¥1,000 book voucher	4000	886	786	22.2%	19.7%	Suzuken (Lifecorder EX)	Worn for ≥10 hours/day for at least 4 days	A stratified random sample was selected from the Basic Resident Register based on age and gender, and individuals were first invited to participate in a survey using a questionnaire, with a request for their responses. At the same time, they were also invited to participate in an additional survey using an accelerometer. Those who agreed to participate in the additional survey were later mailed an accelerometer and a questionnaire.
3	Amagasa et al.	2015	65–74 years	Men, Women	Tokyo, Shizuoka	Stratified random sampling	Mail	Follow-up Invitation	NA	2700	478	450	17.7%	16.7%	Omron Healthcare (Active Style Pro HJA-350IT)	Worn for ≥10 hours/day for at least 4 days	A stratified random sample of elderly individuals was selected from the Basic Resident Register and invited to participate in a mail survey. Those who responded and agreed to follow-up were invited for a follow-up survey by mail several years later, at which point they were also asked to participate in an accelerometer-based survey.
4	Harada et al.	2016	53–70 years	Men, Women (couple)	Hyogo	Random sampling	Mail	Direct Mail	NA	1080	158	153	14.6%	14.2%	Omron Healthcare (Active Style Pro HJA-750C)	Worn for ≥10 hours/day for at least 4 days	Men and their spouses were randomly selected from the Basic Resident Register. A total of 540 couples were invited to participate in a mail survey, which included a questionnaire and an accelerometer-based survey.
5	Shibata et al.	2013–2015	40–60 years	Men, Women	Ehime, Tokyo	Stratified random sampling	Mail	Direct Mail	¥1,000 book voucher	6000	779	711	13.0%	11.9%	Omron Healthcare (Active Style Pro HJA-350IT)	Worn for ≥10 hours/day for at least 4 days	A stratified random sample was selected from the Basic Resident Register based on age and gender, and individuals were invited to participate in the study. Those who agreed to participate were mailed a questionnaire and an accelerometer.

References

1. Kondo K, Lee JS, Kawakubo K, Kataoka Y, Asami Y, Mori K, et al. Association between daily physical activity and neighborhood environments. Environ Health Prev Med. 2009;14(3):196-206. doi: 10.1007/s12199-009-0081-1. PMID: 19568848
2. Inoue S, Ohya Y, Odagiri Y, Takamiya T, Kamada M, Okada S, et al. Characteristics of accelerometry respondents to a mail-based surveillance study. J Epidemiol. 2010;20(6):446-452. doi: 10.2188/jea.je20100062. PMID: 20877141
3. Amagasa S, Inoue S, Murayama H, Fujiwara T, Kikuchi H, Fukushima N, et al. Accelerometer-assessed physical activity among older Japanese adults in rural area: NEIGE study. Res Exer Epidemiol. 2021;23(2):200-201. doi: 10.24804/ree.2124. (in Japanese)
4. Harada K, Masumoto K, Kondo N. Accelerometer-assessed physical activity among community-dwelling middle-aged and older Japanese adults: survey on daily life of

middle-aged and older couples. Res Exer Epidemiol. 2021;23(2):198-199. doi: 10.24804/ree.2119. (in Japanese)

5. Shibata A, Ishii K, Oka K. Accelerometer-assessed physical activity and sedentary behavior among community-dwelling middle-aged Japanese adults: Matsuyama-Koto Study. Res Exer Epidemiol. 2022;24(2):116-117. doi: 10.24804/ree.2138. (in Japanese)
