# Supplementary material for: Physical activity and sedentary behavior surveillance using accelerometers in Japanese urban adults: A descriptive study of participation and adherence
Source: PLoS One. 2026 Jun 1;21(6):e0350144. doi: 10.1371/journal.pone.0350144 (PMC13225377; doi:10.1371/journal.pone.0350144)
Supplement: S3 Table — aParticipants with at least four valid days (including one weekend). All characteristics were included in the model simultaneously. Bold values indicate P < 0.05. (PDF) [file pone.0350144.s003.pdf]

S3 Table. Factors associated with participating in the survey and adherence to valid wear.

Variables	Participation (n = 205)				Adherence (n = 189)[a]			
	Prevalence ratio	95% CI			Prevalence ratio	95% CI		
		Lower	–	Upper		Lower	–	Upper
Age								
20–39 years	Reference				Reference			
40–59 years	**1.79**	**1.30**	**–**	**2.46**	**2.00**	**1.41**	**–**	**2.86**
60–70 years	**1.44**	**1.01**	**–**	**2.05**	**1.65**	**1.12**	**–**	**2.42**
Gender								
Men	Reference				Reference			
Women	1.03	0.82	–	1.28	1.07	0.84	–	1.35
Population								
< 0.1 million	Reference				Reference			
≥ 0.1 million	**0.69**	**0.53**	**–**	**0.90**	**0.68**	**0.52**	**–**	**0.90**
≥ 0.3 million	**0.74**	**0.56**	**–**	**0.99**	**0.72**	**0.53**	**–**	**0.97**

[a]Participants with at least four valid days (including one weekend).

All characteristics were included in the model simultaneously.

Bold values indicate $P < 0.05$.
