# Supplementary material for: Physical activity and sedentary behavior surveillance using accelerometers in Japanese urban adults: A descriptive study of participation and adherence
Source: PLoS One. 2026 Jun 1;21(6):e0350144. doi: 10.1371/journal.pone.0350144 (PMC13225377; doi:10.1371/journal.pone.0350144)
Supplement: S4 Table — LPA, light-intensity physical activity; MHLW, Ministry of Health, Labour and Welfare; MVPA, moderate-to-vigorous physical activity; SB, sedentary behavior. a ≥ 60 min/day of MVPA for 20–64 years and ≥ 40 min/day of MVPA for 65 years and older. (PDF) [file pone.0350144.s004.pdf]

S4 Table. Descriptive statistics of the accelerometer-measured physical activity and sedentary behavior in the participants who adhered to the valid wear (*n* = 189).

Characteristics	Overall (*n* = 189)				By age: 20–64 years (*n* = 142)				By age: ≥ 65 years (*n* = 47)				By gender: Men (*n* = 96)				By gender: Women (*n* = 93)			
	Median	Min	–	Max	Median	Min	–	Max	Median	Min	–	Max	Median	Min	–	Max	Median	Min	–	Max
Wearing day, days	7	4	–	14	7	4	–	14	7	5	–	11	7	4	–	14	7	4	–	12
Wear time, min/day	926.8	675.4	–	1292.6	955.3	675.4	–	1292.6	903.6	702.4	–	1218.1	915.0	700.2	–	1221.7	957.3	675.4	–	1292.6
Time-use, min/day																				
SB	529.9	135.5	–	900.9	538.6	135.5	–	900.9	492.6	186.9	–	776.1	558.6	135.5	–	900.9	500.9	186.9	–	850.7
LPA	330.4	94.6	–	628.8	333.0	94.6	–	628.8	322.7	155.3	–	628.5	290.1	94.6	–	526.5	390.1	157.1	–	628.8
MVPA	56.0	2.7	–	212.1	56.9	5.4	–	212.1	45.9	2.7	–	164.9	57.4	5.4	–	212.1	55.0	2.7	–	189.6
Proportion of wear time, %																				
SB	57.6	18.0	–	86.4	57.4	18.0	–	86.4	57.9	21.7	–	81.6	60.9	18.0	–	86.4	52.3	21.7	–	77.2
LPA	36.2	12.1	–	65.0	36.0	12.1	–	61.9	37.1	16.3	–	65.0	32.2	12.1	–	61.7	41.0	19.2	–	65.0
MVPA	6.2	0.3	–	27.7	6.2	0.5	–	27.7	5.5	0.3	–	21.7	6.2	0.5	–	27.7	6.1	0.3	–	21.3
Step count, steps/day	6925	1299	–	18853	7033	1299	–	18853	5693	1854	–	14872	7046	1299	–	18853	6699	1497	–	14872
	n		%		*n*		%		*n*		%		*n*		%		*n*		%	
Met the MHLW guideline,[a] *n*(%)	95		50.3		66		46.5		29		61.7		52		54.2		43		46.2	

LPA, light-intensity physical activity; MHLW, Ministry of Health, Labour and Welfare; MVPA, moderate-to-vigorous physical activity; SB, sedentary behavior.

[a]≥ 60 min/day of MVPA for 20–64 years and ≥ 40 min/day of MVPA for 65 years and older.
