# Supplementary material for: Physical activity and sedentary behavior surveillance using accelerometers in Japanese urban adults: A descriptive study of participation and adherence
Source: PLoS One. 2026 Jun 1;21(6):e0350144. doi: 10.1371/journal.pone.0350144 (PMC13225377; doi:10.1371/journal.pone.0350144)
Supplement: S5 Table — All characteristics were included in the model simultaneously. Bold values indicate P < 0.05. (PDF) [file pone.0350144.s005.pdf]

S5 Table. Factors associated with the reasons for nonparticipation in the survey among the invited sample (*n* = 650).

Variables	No contact/pretending absent				Refusal (inconvenience/busy)				Difficulty (accelerometer issues)				Difficulty (questionnaire issues)			
	Prevalence ratio	95% CI			Prevalence ratio	95% CI			Prevalence ratio	95% CI			Prevalence ratio	95% CI		
		Lower	–	Upper		Lower	–	Upper		Lower	–	Upper		Lower	–	Upper
Age																
20–39 years	Reference				Reference				Reference				Reference			
40–59 years	**0.59**	**0.43**	**–**	**0.82**	0.89	0.70	–	1.15	1.02	0.48	–	2.18	1.08	0.20	–	5.79
60–70 years	**0.33**	**0.21**	**–**	**0.52**	1.21	0.95	–	1.54	1.36	0.64	–	2.89	2.35	0.46	–	11.99
Gender																
Men	Reference				Reference				Reference				Reference			
Women	0.92	0.68	–	1.25	1.07	0.89	–	1.29	0.96	0.54	–	1.72	0.35	0.10	–	1.24
Population																
< 0.1 million	Reference				Reference				Reference				Reference			
≥ 0.1 million	1.26	0.84	–	1.90	1.20	0.94	–	1.55	0.77	0.37	–	1.59	4.33	0.53	–	35.41
≥ 0.3 million	1.29	0.84	–	1.96	1.11	0.84	–	1.45	1.09	0.52	–	2.28	2.25	0.24	–	21.27

All characteristics were included in the model simultaneously.

Bold values indicate $P < 0.05$.
