# Supplementary material for: Physical activity and sedentary behavior surveillance using accelerometers in Japanese urban adults: A descriptive study of participation and adherence
Source: PLoS One. 2026 Jun 1;21(6):e0350144. doi: 10.1371/journal.pone.0350144 (PMC13225377; doi:10.1371/journal.pone.0350144)
Supplement: S6 Table — PA, physical activity; SB, sedentary behavior. All characteristics were included in the model simultaneously. Bold values indicate P < 0.05. (PDF) [file pone.0350144.s006.pdf]

S6 Table. Factors associated with the reasons for participation in the survey among the participants who adhered to the valid wear (*n* = 189).

Variables	Interest in PA and SB				Clear explanation by investigators				Desire for societal contribution				Health consciousness				Incentive for participation			
	Prevalence ratio	95% CI			Prevalence ratio	95% CI			Prevalence ratio	95% CI			Prevalence ratio	95% CI			Prevalence ratio	95% CI		
		Lower	–	Upper		Lower	–	Upper		Lower	–	Upper		Lower	–	Upper		Lower	–	Upper
Age																				
20–39 years	Reference				Reference				Reference				Reference				Reference			
40–59 years	1.93	0.72	–	5.16	1.00	0.44	–	2.27	1.21	0.62	–	2.34	1.98	0.63	–	6.24	0.78	0.59	–	1.03
60–70 years	**3.27**	**1.22**	–	**8.81**	1.05	0.43	–	2.53	1.55	0.79	–	3.04	**4.72**	**1.55**	–	**14.39**	**0.41**	**0.26**	–	**0.65**
Gender																				
Men	Reference				Reference				Reference				Reference				Reference			
Women	**1.77**	**1.11**	–	**2.82**	1.41	0.81	–	2.44	1.03	0.69	–	1.53	1.43	0.91	–	2.24	0.81	0.61	–	1.06
Population																				
< 0.1 million	Reference				Reference				Reference				Reference				Reference			
≥ 0.1 million	0.86	0.50	–	1.45	1.57	0.79	–	3.13	1.15	0.70	–	1.88	1.37	0.75	–	2.51	0.93	0.66	–	1.32
≥ 0.3 million	0.82	0.46	–	1.45	1.29	0.61	–	2.71	1.14	0.68	–	1.93	1.47	0.79	–	2.75	1.09	0.79	–	1.51

PA, physical activity; SB, sedentary behavior.

All characteristics were included in the model simultaneously. Bold values indicate $P < 0.05$.
